# Application of Fractal Radiomics and Machine Learning for Differentiation of Non-Small Cell Lung Cancer Subtypes on PET/MR Images

**DOI:** 10.3390/jcm14165776

**Published:** 2025-08-15

**Authors:** Ewelina Bębas, Konrad Pauk, Jolanta Pauk, Kristina Daunoravičienė, Małgorzata Mojsak, Marcin Hładuński, Małgorzata Domino, Marta Borowska

**Affiliations:** 1 Institute of Biomedical Engineering, Bialystok University of Technology, 15-351 Białystok, Poland; ewebeb7@gmail.com (E.B.); j.pauk@pb.edu.pl (J.P.); 2 Faculty of Medicine, Warsaw Medical University, 02-091 Warszawa, Poland; konrad.pauk@gmail.com; 3 Department of Biomechanical Engineering, Vilnius Gediminas Technical University, 10223 Vilniaus, Lithuania; kristina.daunoraviciene@vlniustech.lt; 4 Laboratory of Molecular Imaging, Medical University of Białystok, 15-089 Białystok, Poland; malgorzata.mojsak@umb.edu.pl (M.M.); hladunski.marcin@gmail.com (M.H.); 5 Department of Large Animal Diseases and Clinic, Institute of Veterinary Medicine, Warsaw University of Life Science, 02-787 Warszawa, Poland

**Keywords:** adenocarcinoma, squamous cell carcinoma, magnetic resonance, fractal analysis, texture analysis

## Abstract

**Objectives:** Non-small cell lung cancer (NSCLC), the most prevalent type of lung cancer, includes subtypes such as adenocarcinoma (ADC) and squamous cell carcinoma (SCC), which require distinct management approaches. Accurately differentiating NSCLC subtypes based on diagnostic imaging remains challenging. However, the extraction of radiomic features—such as first-order statistics (FOS), second-order statistics (SOS), and fractal dimension texture analysis (FDTA) features—from magnetic resonance (MR) images supports the development of quantitative NSCLC assessments. **Methods:** This study aims to evaluate whether the integration of FDTA features with FOS and SOS texture features in MR image analysis improves machine learning classification of NSCLC into ADC and SCC subtypes. The study was conducted on 274 MR images, comprising ADC (n = 122) and SCC (n = 152) cases. From the segmented MR images, 93 texture features were extracted. The random forest algorithm was used to identify informative features from both FOS/SOS and combined FOS/SOS/FDTA datasets. Subsequently, the k-nearest neighbors (kNN) algorithm was applied to classify MR images as ADC or SCC. **Results:** The highest performance (accuracy = 0.78, precision = 0.81, AUC = 0.89) was achieved using 37 texture features selected from the combined FOS/SOS/FDTA dataset. **Conclusions:** Incorporating fractal descriptors into the texture-based classification of lung MR images enhances the differentiation of NSCLC subtypes.

## 1. Introduction

Lung cancer remains the leading cause of cancer-related mortality worldwide, with non-small cell lung cancer (NSCLC) accounting for approximately 85% of all cases [1]. In Poland, around 20,000 new NSCLC cases are diagnosed annually, with mortality rates nearly matching incidence due to the predominance of late-stage detection [2]. NSCLC is associated with well-established risk factors, including chronic tobacco exposure, occupational inhalation of asbestos and heavy metals, exposure to residential radon gas, and genetic polymorphisms that impair detoxification or DNA repair pathways [3]. NSCLC represents a heterogeneous disease comprising several specific subtypes, with adenocarcinoma (ADC) and squamous cell carcinoma (SCC) being the most common [4,5]. ADC typically arises in the peripheral regions of the lungs and is more prevalent among never-smokers, while SCC is usually centrally located and strongly linked to smoking [4]. Given that treatment protocols for NSCLC subtypes differ, accurate differentiation between ADC and SCC is crucial for improving survival outcomes and reducing mortality rates. For instance, ADC harboring *EGFR* or *ALK* mutations may respond well to tyrosine kinase inhibitors, whereas PD-L1-positive SCC may benefit from immunotherapy [5].

The differential diagnosis of NSCLC is primarily based on histological classification supported by molecular profiling, with tissue samples typically obtained via bronchoscopy-guided biopsy or ultrasound-guided transthoracic biopsy. Histological and molecular confirmation of NSCLC subtypes remains the diagnostic gold standard [3,4]. However, each biopsy procedure is invasive, and even when performed correctly, may yield insufficient tissue for comprehensive analysis. Therefore, increasing efforts are being made to develop and integrate non-invasive diagnostic modalities—particularly advanced imaging techniques—into the NSCLC diagnostic protocol.

Lung cancer diagnostic protocols generally include imaging modalities such as conventional radiography, computed tomography (CT), and/or magnetic resonance (MR) imaging of the chest [6,7]. Due to its ability to accurately image both bone and air-filled structures and its superior spatial resolution compared to conventional radiography, CT remains central to the initial recognition and screening of NSCLC [6]. Meanwhile, MR imaging has emerged as a promising radiation-free alternative, offering superior soft tissue contrast [7,8]. However, despite the advantages of CT and MR three-dimensional imaging, visual radiological evaluation alone does not enable reliable distinction between NSCLC subtypes, which is essential for guiding appropriate treatment. As a result, the next advancement in lung imaging was the introduction of positron emission tomography (PET), particularly using ^18^F-fluorodeoxyglucose, which offers functional insight into tumor metabolism and serves as a surrogate marker for malignancy and aggressiveness [9].

Unfortunately, hybrid imaging modalities that fuse metabolic and anatomical data, such as PET/CT and PET/MR, are limited by their high cost and restricted availability [10,11]. Consequently, an alternative step in the evolution of advanced lung imaging has been the enhancement of CT and MR image interpretation through radiomics analysis. Radiomics enables the extraction and quantification of high-dimensional features from medical images using data characterization algorithms [12]. These features include intensity, shape, texture, and wavelet-based descriptors, which have shown promise in assessing NSCLC heterogeneity, often correlating well with clinical markers and gene expression profiles. Among texture-based features, first-order statistics (FOS) and second-order statistics (SOS)—which describe intensity distributions and spatial relationships among pixels—have been successfully applied [12]. However, lung image textures may also contain additional meaningful patterns rooted in fractal geometry. Fractal-based features, such as multiresolution fractal feature vectors, could play a critical role in improving the imaging-based characterization of NSCLC.

The fractal feature vector represents a novel approach for quantifying structural complexity in biological tissues through fractal dimension texture analysis (FDTA). Many anatomical structures—including the bronchial tree, vascular networks, and alveolar architecture—exhibit fractal geometry. As such, FDTA features may capture this complexity and have demonstrated diagnostic relevance in various cancers, including brain, breast, and ovarian malignancies [13,14]. Initial attempts to apply fractal feature vectors in lung MR image analysis were limited by motion artifacts and inadequate resolution [15]. However, the development of high-resolution, motion-compensated MR imaging of the chest [16] now provides the technical foundation for reliably extracting and quantifying geometry-specific features from NSCLC images using FDTA. We hypothesize that FDTA features derived from high-resolution lung MR images can enhance the non-invasive differentiation of NSCLC subtypes. This study aims to evaluate whether the integration of FDTA features into FOS/SOS texture analysis of MR images can improve machine learning-based classification of NSCLC into ADC and SCC subtypes.

## 2. Materials and Methods

### 2.1. Study Design

**Figure 1:**
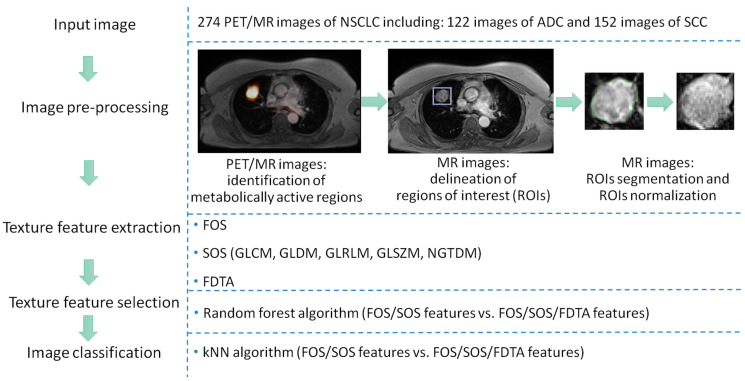
The study workflow on PET/MR images of non-small cell lung cancer (NSCLC) including both adenocarcinoma (ADC) and squamous cell carcinoma (SCC). Images were pre-processed to segment regions of interest (ROIs). From given ROIs, texture features were extracted using first-order statistics (FOS), second-order statistics (SOS), and fractal dimension texture analysis (FDTA). Informative texture features were selected using random forest algorithm. Based on the selected texture features, images were classified using k-nearest neighbors (kNN) algorithm.

The study was designed to utilize PET/MR input images to differentiate NSCLC subtypes based on radiomic features extracted from MR images. PET images were used for the initial identification of metabolically active regions, after which the corresponding MR images were segmented and further analyzed. The study workflow is presented in Figure 1 and includes the following steps performed on the input images: image pre-processing, texture feature extraction, texture feature selection, and image classification.

### 2.2. Subjects

The study was conducted on a total of 274 PET/MR images collected from 45 patients (n = 45; mean age: 65 years; range: 35–82 years; 32 men and 13 women) with lung cancer stage I or II histologically confirmed NSCLC after surgery. The initial diagnosis, depending on the location of the lesion suspected of being proliferative, was based on microscopic examination, transthoracic needle biopsy, examination of samples taken during bronchoscopy, or mediastinoscopy. A total of 122 PET/MR images were obtained from 24 patients with ADC (n = 24; mean age: 64 ± 9 years; 14 men and 10 women), while 152 PET/MR images were collected from 21 patients with SCC (n = 21; mean age: 66 ± 6 years; 18 men and 3 women). Inclusion criteria were age above 18 years, histologically confirmed diagnosis of ADC or SCC, and ability to hold breath during scanning. Exclusion criteria included: coexisting severe lung disease, MR-unsafe implants, pregnancy, or breastfeeding.

### 2.3. Input Images

Images were collected using a hybrid 3T PET/MR system (MAGNETOM Biograph, Siemens Healthineers, Forchheim, Germany), enabling the simultaneous acquisition of anatomical (MR) and metabolic (PET) data. This dual-modality setup allowed for image fusion and integrated structural–functional assessment. Patients were positioned using stabilizing supports, and motion correction algorithms were applied to minimize motion artifacts and geometric distortions. The MR imaging protocol included two sequences: T2-weighted Half Fourier Acquisition Single-shot Turbo Spin Echo (HASTE) for high-resolution soft tissue assessment (voxel size: 1.0 × 1.0 × 6.0 mm; TR: 1500 ms), and T1-weighted Volumetric Interpolated Breath-hold Examination (VIBE) for detailed morphological imaging of the lungs (voxel size: 1.6 × 1.6 × 4.0 mm; TR: 4.02 ms). The PET imaging protocol involved the administration of the radiotracer ^18^F-fluorodeoxyglucose to assess metabolic activity within tumor tissue. The radiotracer dose was individually adjusted based on patient body weight, in accordance with clinical PET imaging guidelines, and ranged from 280 to 350 MBq. PET images were acquired 60 min post-injection.

### 2.4. Image Pre-Processing

Metabolically active regions were identified on PET/MR images using the standardized uptake value (SUVmax), which was 8.03 ± 4.06 for ADC images and 11.86 ± 3.42 for SCC images. Based on these values, NSCLC lesions were initially localized in both upper and lower lung lobes, presenting as tumors of varying sizes (ADC: shortest diameter 31.9 ± 14.4 mm, longest diameter 42.1 ± 21.2 mm; SCC: shortest diameter 38.9 ± 11.7 mm, longest diameter 52.8 ± 17.6 mm).

**Figure 2:**
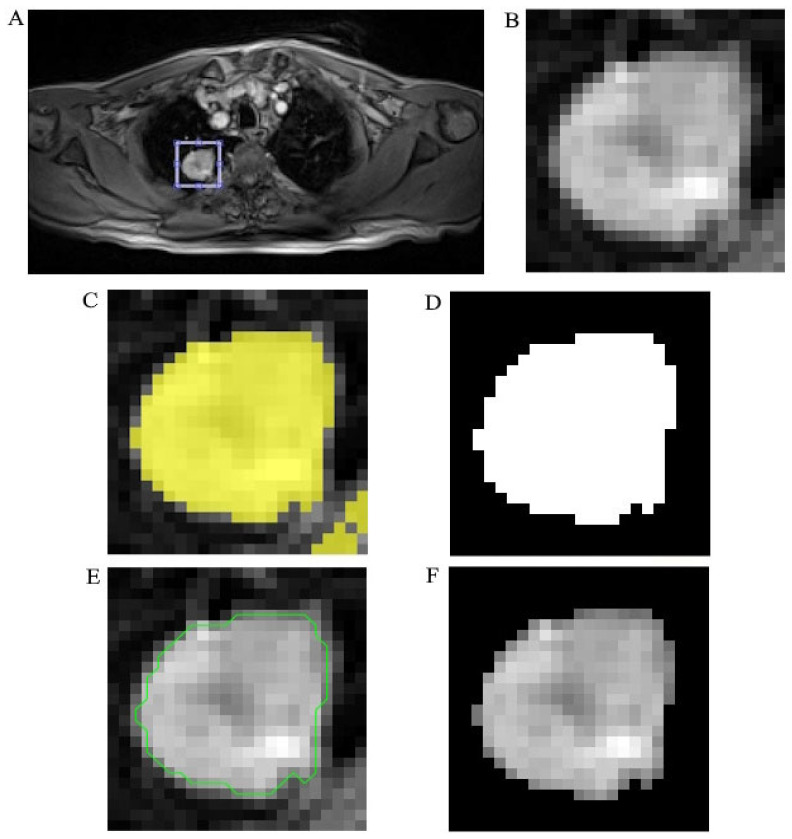
The segmentation process of MR image. (**A**) Non-small cell lung cancer (NSCLC) localization on input MR image. (**B**) Delineation of the region of interest (ROI). (**C**) Thresholding and binarization. (**D**) Resulting binary masks. (**E**) Contour enhancing (green line). (**F**) Final segmented ROI exporting.

In the identified regions, NSCLC segmentation was performed semi-automatically on MR images using the Image Segmenter tool in MATLAB version R2023a and the SimpleITK library in Python version 3.11. The segmentation process included the following steps: DICOM image input, grayscale conversion, delineation of regions of interest (ROIs), thresholding and binarization, contour enhancement, and export of segmented ROIs. ROIs were manually delineated by two independent radiologists, and any discrepancies were resolved by consensus. To distinguish NSCLC regions from surrounding healthy tissue, Otsu’s thresholding algorithm [17] was applied. This data-driven method, based on histogram shape analysis, enables automatic image binarization. The resulting binary masks were validated by two experienced radiologists. Further refinement of the binary masks was performed using morphological operations—including dilation, erosion, opening, and closing—to enhance spatial coherence and contour accuracy. Final segmentation masks were exported in NIfTI format for compatibility with radiomics workflows and were overlaid on grayscale MR images for visual confirmation of tumor boundaries. An example of the MR image segmentation process is presented in Figure 2.

Following segmentation, noise reduction was applied to the MR images to improve the quality of feature extraction. This filtering process was implemented using the SimpleITK library in Python [18]. A normalization filter was applied to rescale voxel intensities into a standardized range, thereby reducing acquisition-dependent variability and enhancing the reproducibility of radiomic descriptors [19,20].

### 2.5. Texture Feature Extraction

Texture feature extraction was performed on the normalized MR dataset using the PyRadiomics package version 3.1.0 in Python [21]. All texture features were derived from the segmented binary ROIs. Three approaches were employed for texture feature extraction: FOS, SOS, and FDTA. The FOS approach yielded 17 features. SOS features were extracted using five methods, producing a total of 73 features:

- Gray-Level Co-occurrence Matrix (GLCM)—22 features,
- Gray-Level Dependence Matrix (GLDM)—14 features,
- Gray-Level Run Length Matrix (GLRLM)—16 features,
- Gray-Level Size Zone Matrix (GLSZM)—16 features,
- Neighboring Gray-Tone Difference Matrix (NGTDM)—5 features,

The FDTA approach generated 3 features. In total, 93 features were extracted from each ROI and included in further analysis. A summary of the extracted texture features is presented in Table 1.

#### Fractal Dimension Texture Analysis

The FDTA [22] is based on the Fractional Brownian Motion (FBM) model [23], which characterizes the roughness of natural surfaces as a result of a random walk. Features used to represent the complexity and self-similarity of NSCLC architecture as a fractal surface include the fractal dimension (FD), lacunarity, and the Fractal Scale Variability Index (FSVI).

FD is a key feature of fractal geometry, which can be estimated from Equation (1):

(1) $$E\left({∆I_{\vec{r}}}^{2}\right)=c{\left(∆\vec{r}\right)}^{6-2FD}$$

where E(·) means the expectation operator, $\vec{r}=(x,y)$—the position in two dimensional space, $I_{\vec{r}}$—the intensity of pixel at position $\vec{r}$, $∆\vec{r}=\sqrt{{\left(x_{2}-x_{1}\right)}^{2}+{\left(y_{2}-y_{1}\right)}^{2}}$—the spatial distance between two pixels $\left(x_{1},y_{1}\right)$ and $\left(x_{2},y_{2}\right)$, $∆I_{\vec{r}}=I\left(\vec{r}+∆\vec{r}\right)-I(\vec{r})$ is the intensity variations between two pixels, c—constant.

More simply, the Hurst coefficient (H) can firstly be determined from Equation (2):

(2) $$E\left(\left|∆I_{\vec{r}}\right|\right)=k{\left(∆\vec{r}\right)}^{H}$$

where $k=\;{E\left(\left|∆I_{\vec{r}}\right|\right)}_{∆\vec{r}=1}$, and taking the log function to both sides of Equation (3) can be obtained:

(3) $$\log E\left(\left|∆I_{\vec{r}}\right|\right)=\log k+H\cdot \log∆\vec{r}$$

Then, FD can be easily calculated, using Equation (3) of the H parameter estimation, from Equation (4):

(4) FD=3−H

H is the constant, ranging from 0 to 1. A small value of FD (corresponding to a large value of H) means a smooth surface. In contrast, a large value of FD (corresponding to a small value of H) indicates a rough surface.

In realization of this algorithm for a given N×N image, the average of the absolute intensity difference of all pixel pairs with vertical or horizontal distance k is defined as follows from Equation (5):

(5) $$ID\left(k\right)=\frac{\sum_{r=0}^{N-k-1}\left|I\left(\vec{r}+∆\vec{r}\right)-I(\vec{r})\right|}{2N(N-k-1)\;}$$

The multiscale intensity difference vector (MIDV) is defined as (6):

(6) $$IDV=[ID\left(1\right),\;ID\left(2\right),\;\ldots ,\;ID(s)]$$

where s is the maximum possible scale.

The value H can be obtained by using least squares linear regression to estimate the slope of the curve of ID(k) versus k in log-log scale.

Based on the concept of the H parameter estimation, the multiresolution fractal (MF) feature vector can be defined as (7):

(7) $$MF=[H^{n},\;H^{n-1},\ldots ,H^{n-m-1}]$$

where $N=2^{n}$ is the size of the original image, $H^{k}$ is the H parameter of the image $I^{k}$, and m is the number of resolution level.

The MF vector represents a set of descriptors that reflect the roughness or smoothness of a texture at multiple resolutions. These descriptors are the basis for the definition of a novel FSVI feature that examines the strength and direction of the relationship between resolution m and the H parameter, defined as (8):

(8) $$FSVI=\frac{m\sum_{i=0}^{m-1}i\cdot H_{i}-\sum_{i=0}^{m-1}i\cdot \sum_{i=0}^{m-1}H_{i}}{m\sum_{i=0}^{m-1}i^{2}-{\left(\sum_{i=0}^{m-1}i\right)}^{2}}$$

where i is the index of resolution levels, running from i=0,1,…,m−1, with m denoting the maximum number of resolutions, and $H_{i}$ is the Hurst exponent calculated at the corresponding resolution i.

In addition to the FD and Hurst exponent, lacunarity describes the spatial heterogeneity of an image—that is, the distribution of “gaps” within its structure [23]. Two images can have the same FD (indicating similar roughness or complexity across scales), but one may exhibit a homogeneous texture with low lacunarity, where the structure fills space uniformly, while the other shows irregular clusters, local density variations, and holes, corresponding to high lacunarity.

One of the classic ways of calculating lacunarity is the box-counting method. In a simple way, the lacunarity Λ for a box of size l is defined as (9):

(9) $$\Lambda \left(l\right)=\frac{\sum_{l=1}^{L}l^{2}\cdot P(l)-{\left(\sum_{l=1}^{L}l\cdot P(l)\right)}^{2}}{{\left(\sum_{l=1}^{L}l\cdot P(l)\right)}^{2}}$$

where P( l ) is the probability that there are l points within a box of size A centered about an arbitrary point of O; L refers to the total number of possible point counts within a box, and $\sum_{l=1}^{L}P(l)=1$.

### 2.6. Texture Feature Selection

Texture feature selection was performed using the PyRadiomics package version 3.1.0 in Python [21]. Features were selected using the random forest algorithm applied to two combined texture feature datasets: one containing FOS and SOS features (FOS/SOS dataset, n = 199), and the other containing FOS, SOS, and FDTA features (FOS/SOS/FDTA dataset, n = 202). A random forest model with 200 estimators (decision trees) was employed to evaluate the significance of the features. The algorithm was calibrated using a fixed random seed (random_state = 42) to ensure reproducibility. After training, each feature was assigned an importance score reflecting its impact on the classification outcome.

### 2.7. Image Classification

Image classification was implemented using the k-nearest neighbors (kNN) algorithm, a supervised machine learning method, and the Scikit-learn library in Python [18]. The kNN algorithm assumes that objects close to each other in feature space are likely to belong to the same class. For classification, the algorithm assigns a new data point to the class most common among its k = 7 nearest neighbors in the feature space [24,25]. Classification was performed separately using three datasets:

- 37 texture features that showed significant differences between ADC and SCC.
- Up to 40 texture features selected from the FOS/SOS dataset.
- Up to 40 texture features selected from the FOS/SOS/FDTA dataset.

Images were classified as 0 (ADC) or 1 (SCC) based solely on the selected texture features extracted from MR images. Classification metrics were calculated relative to the histological confirmation of the NSCLC subtypes. For both datasets, the following classification metrics were computed: accuracy, precision, recall, F1 score, and the area under the curve (AUC) of the receiver operating characteristic (ROC). Classification performance was evaluated using 5-fold stratified cross-validation accuracy (CV accuracy).

### 2.8. Statistical Analysis

Statistical analysis was performed using GraphPad Prism, version 6 (GraphPad Software Inc., San Diego, CA, USA). Each texture feature extracted from MR images was treated as a data series and grouped by ADC and SCC subtypes. Data distribution was assessed using the Shapiro–Wilk test. Since not all data series were normally distributed, results are presented as median and range (lower quartile (Q1) and upper quartile (Q3)).

For each texture feature, comparisons between ADC and SCC groups were made using an unpaired *t*-test with Welch’s correction when both groups were normally distributed. If at least one group did not follow a normal distribution, the non-parametric Mann–Whitney test was applied. Statistical significance was defined as *p* < 0.05.

## 3. Results

### 3.1. Texture Feature Characteristics Among NSCLC Subtypes

Among the 93 texture features extracted using the three approaches—FOS, SOS, and FDTA—37 features showed significant differences between ADC and SCC. Values for all studied texture features are summarized in Supplementary Table S1–S7.

For the FOS features, 11 of 17 (64.7%) differed between ADC and SCC. Mean, median, 90th percentile, interquartile range (IR), median absolute deviation (MAD), robust MAD (rMAD), root mean square (RMS), and variance were higher in ADC MR images than in SCC images. In contrast, Kurtosis, Minimum, and 10th Percentile were lower in ADC compared to SCC (Table 2).

Regarding SOS features extracted using the GLCM, only 4 of 22 (18.2%) differed between ADC and SCC. Cluster Prominence (CP), Correlation, and Informational Measure of Correlation 2 (IMC2) were higher in ADC, while Cluster Shade (CS) was lower compared to SCC (Table 3).

For SOS features extracted using the GLDM, only 1 of 14 (7.1%) differed between ADC and SCC. Small Dependence High Gray-Level Emphasis (SDHGLE) was lower in ADC than in SCC (Table 4).

Among SOS features extracted using the GLRLM, 7 of 16 (43.8%) differed. Gray-Level Non-Uniformity (GLNN) and Long Gray-Level Run Emphasis (LGLRE) were higher in ADC, whereas Short Run Emphasis (SRE), Gray-Level Variance (GLV), High Gray-Level Run Emphasis (HGLRE), Short Run Low Gray-Level Emphasis (SRLGLE), and Short Run High Gray-Level Emphasis (SRHGLE) were lower compared to SCC (Table 5).

For SOS features extracted using the GLSZM, 10 of 16 (62.5%) showed differences. Size Zone Non-Uniformity (SZNN), Gray-Level Variance (GLV), and High Gray-Level Zone Emphasis (HGLZE) were higher in ADC, while Gray-Level Non-Uniformity (GLN), Gray-Level Non-Uniformity Normalized (GLNN), Size Zone Non-Uniformity (SZN), Zone Percentage (ZP), Zone Entropy (ZE), Low Gray-Level Zone Emphasis (LGLZE), and Small Area Low Gray-Level Emphasis (SALGLE) were lower compared to SCC (Table 6).

Considering SOS features extracted using the NGTDM, 2 of 5 (40.0%) differed. Both Coarseness and Strength were higher in ADC than in SCC (Table 7).

Among FDTA features, 2 of 3 (66.7%) differed between ADC and SCC. The FD was lower, while lacunarity was higher in ADC compared to SCC (Table 8).

### 3.2. Texture Feature Selection for the NSCLC Subtype Classification

**Figure 3:**
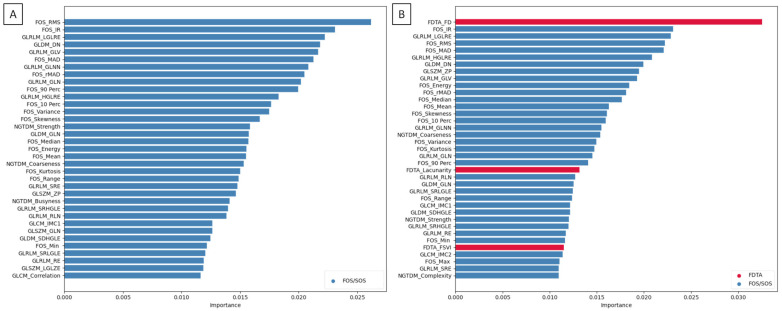
The importance of texture features selected for the non-small cell lung cancer (NSCLC) subtype classification based on MR images. Texture features selected from (**A**) FOS/SOS dataset and (**B**) FOS/SOS/FDTA dataset using the random forest algorithm. Selected FDTA features are additionally marked in red.

From the FOS/SOS dataset, 35 texture features were selected for NSCLC subtype classification. The importance of these features is presented in Figure 3A. Notably, this selection excluded some GLCM-derived features (CP, CS, IMC2) and GLSZM-derived features (GLNN, SZN, SZNN, GLV, ZE, HGLZE, SALGLE), despite their significant differences between ADC and SCC MR images. However, all significantly different features derived from FOS, GLDM, GLRLM, and NGTDM were included. In addition, several features that did not show statistically significant differences between ADC and SCC—such as FOS-derived features (Skewness, Energy, Range), a GLCM-derived feature (IMC1), GLDM-derived features (GLN, DN), GLRLM-derived features (GLN, RLN, RE), and an NGTDM-derived feature (Busyness)—were also retained.

From the FOS/SOS/FDTA dataset, 37 texture features were selected for NSCLC subtype classification. The importance of these features is shown in Figure 3B. This set did not include some GLCM-derived features (CP, CS, Correlation) and GLSZM-derived features (GLN, GLNN, SZN, SZNN, GLV, ZE, LGLZE, HGLZE, SALGLE), even though these also differed significantly between ADC and SCC. Nonetheless, all significantly different features from FOS, GLDM, GLRLM, NGTDM, and FDTA were retained. Moreover, some features that did not show statistically significant differences—such as FOS-derived features (Skewness, Energy, Max, Range), a GLCM-derived feature (IMC1), GLDM-derived features (GLN, DN), GLRLM-derived features (GLN, RLN, RE), an NGTDM-derived feature (Complexity), and an FDTA-derived feature (FSVI)—were also included. The texture features used for NSCLC subtype classification are summarized in Supplementary Table S8.

### 3.3. Efficiency of the NSCLC Subtype Classification Based on MR Image Analysis

**Figure 4:**
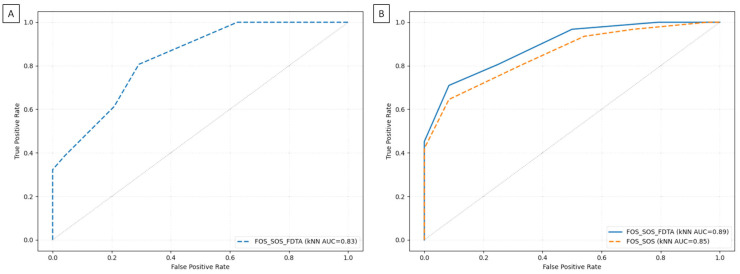
Receiver operating characteristic (ROC) and area under curve (AUC) of the k-nearest neighbors (kNN) algorithm, comparing classification efficiency between the radiomic analysis-based identification and the histological confirmation of the non-small cell lung cancer (NSCLC) subtypes. (**A**) ROC curve returned for texture features from the FOS/SOS/FDTA dataset, which differed significantly between adenocarcinoma (ADC) and squamous cell carcinoma (SCC) MR images. (**B**) ROC curves returned for texture features from the FOS/SOS and FOS/SOS/FDTA datasets, which were selected using the random forest algorithm.

The ROC analysis of the kNN algorithm for classifying NSCLC subtypes demonstrated a high AUC (0.83) when using texture features from the FOS/SOS/FDTA dataset that differed significantly between ADC and SCC MR images; an even higher AUC (0.85) was achieved with the feature set selected from the FOS/SOS dataset; and the highest AUC (0.89) was observed for the feature set selected from the FOS/SOS/FDTA dataset (Figure 4).

**Figure 5:**
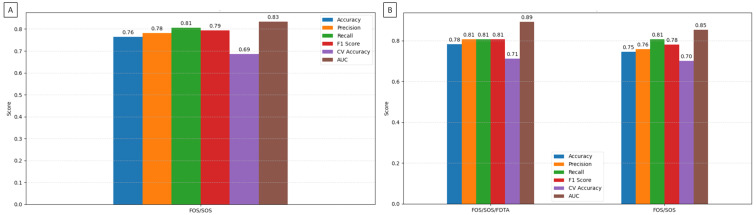
Classification metrics (accuracy, precision, recall, F1 score, and CV accuracy) and area under curve (AUC) of the k-nearest neighbors (kNN) algorithm, comparing classification efficiency between the radiomic analysis-based identification and the histological confirmation of the non-small cell lung cancer (NSCLC) subtypes. (**A**) Classification metrics obtained for texture features from the FOS/SOS/FDTA dataset, which differed significantly between adenocarcinoma (ADC) and squamous cell carcinoma (SCC) MR images. (**B**) Classification metrics obtained for texture features from the FOS/SOS and FOS/SOS/FDTA datasets, which were selected using the random forest algorithm.

These ROC findings were supported by classification metrics, all of which exceeded 0.70 in accuracy. The highest classification performance was achieved by the kNN algorithm using the feature set selected from the FOS/SOS/FDTA dataset, with an accuracy of 0.78, precision of 0.81, and F1 score of 0.81. Slightly lower performance was observed for the FOS/SOS/FDTA dataset containing only features that significantly differed between ADC and SCC (accuracy: 0.76; precision: 0.78; F1 score: 0.79), and the lowest performance was seen with the feature set selected from the FOS/SOS dataset (accuracy: 0.75; precision: 0.76; F1 score: 0.78). CV accuracy was also highest for the feature set selected from the FOS/SOS/FDTA dataset (0.71), followed by the FOS/SOS dataset (0.70), and was lowest for the FOS/SOS/FDTA dataset containing only significantly different features (0.69). For all datasets, recall remained consistent at 0.81 (Figure 5).

## 4. Discussion

SOS-based texture feature analysis is one of the most widely used approaches for structural assessment of medical images [26,27,28,29,30,31,32,33,34,35]. These methods have been applied to CT images for evaluating ADC and SCC architecture [26,27], as well as for NSCLC classification [28,29,30,31,32,33,34,35]. Previous studies have shown that among NSCLC subtypes, ADC displays greater heterogeneity and structural complexity, whereas SCC displays greater homogeneity and more regular cellular architecture [26,27], as demonstrated through texture analysis of PET/CT [26] and CT [27] lung images. Pioneering work by Haralick and Shanmugam [36] first demonstrated that applying SOS approaches to MR images could accurately characterize tumor properties, laying the groundwork for their broad adoption in medical imaging. In this study, the texture of ADC MR images was characterized by greater intensity (e.g., higher mean, MAD, rMAD, RMS, and variance) and the presence of larger, darker pixels (e.g., higher LGLRE and HGLZE), corresponding to increased image heterogeneity (e.g., higher CP and Coarseness). In contrast, the texture of SCC MR images was marked by smaller structures with lighter pixels (e.g., higher SDHGLE, SRLGLE, SRHGLE, SALGLE, and LGLZE), more homogeneous volumes (e.g., higher SZN), and greater overall regularity (e.g., higher CS, SRE, GLN, and GLNN). Importantly, this study also demonstrated for the first time that NSCLC MR images exhibit a fractal structure. ADC was associated with greater heterogeneity and more dispersed space within the fractal structure (higher lacunarity), while SCC showed greater space-filling properties (higher FD) [13]. These FDTA features appear to provide additional insight into tumor architecture, capturing structural heterogeneity and complexity not fully represented by FOS and SOS features alone—ultimately contributing to improved classification performance when included in the analysis.

One may observe that when only FOS/SOS texture features were considered, the most informative features for ADC and SCC subtype differentiation were derived from FOS (RMS and IR), GLRLM (LGLRE and GLV), and GLDM (DN). However, when all FOS/SOS/FDTA texture features were included, the most informative features shifted to those derived from FDTA (FD), FOS (IR, RMS, and MAD), and GLRLM (LGLRE). These results suggest that incorporating FDTA features enhances the discriminatory power between ADC and SCC in MR image-based radiomic analysis. Within the dataset that included FDTA features, the FD exhibited the highest feature importance, indicating that the degree of space-filling in the fractal structure [13] significantly contributes to NSCLC subtype classification. Notably, the remaining two FDTA features—lacunarity and FSVI—were also selected for classification, despite no statistically significant differences in FSVI values between ADC and SCC MR images. This suggests that spatial distribution characteristics, captured by lacunarity [13], and scale-dependent complexity, captured by FSVI [37], may play a meaningful role in distinguishing between ADC and SCC subtypes. While the multiscale FD reflects global geometric complexity, FSVI highlights variations in complexity across different scales. This novel attribute of fractal geometry improves the interpretability of FDTA by identifying scale-dependent irregularities, which may correspond to biologically relevant tumor heterogeneity [38,39]. These findings align with the growing recognition of fractal geometry as a powerful analytical framework for quantifying biological complexity in medical imaging [14,15,38,39].

One may observe that although both classifiers using the FOS/SOS/FDTA datasets included 37 texture features, the specific features differed slightly. This likely explains why the classifier based on the feature-selected FOS/SOS/FDTA dataset outperformed the classifier that used all features from the FOS/SOS/FDTA dataset that differed significantly between ADC and SCC MR images. The random forest algorithm, employed for texture feature selection, is widely regarded as one of the most effective methods for MR image analysis due to its strong capability to assess feature importance, scalability, robustness against overfitting, and ability to account for interactions between features [40,41,42]. The random forest algorithm included all three FDTA features—despite only two showing statistically significant differences between NSCLC subtypes—in the classifier trained on the feature-selected FOS/SOS/FDTA dataset. The inclusion of two, and subsequently all three, FDTA features (including FSVI) led to improved classification performance: more of the overall predictions were correct (accuracy increased from 0.76 to 0.78), and a higher proportion of positive predictions were correct (precision increased from 0.78 to 0.81) [43,44]. However, the inclusion of FSVI features did not affect the recall value, which remained constant at 0.81, indicating that the proportion of actual positives correctly identified was unchanged [43,44]. These findings suggest that incorporating FDTA features into kNN-based classification improves the model’s ability to capture structural heterogeneity relevant to the differentiation of NSCLC subtypes using MR image radiomics.

It is worth noting that the classifier using the FOS/SOS/FDTA dataset with features that significantly differed between ADC and SCC MR images outperformed the classifier based on the feature-selected FOS/SOS dataset, which did not include any FSVI features. This suggests that the inclusion of FSVI features generally improved classification performance, achieving up to 0.78 accuracy and 0.89 AUC using the kNN algorithm. In our previous study, which utilized GLCM and histogram of oriented gradients (HOG) features, the kNN classifier achieved 0.71 accuracy in distinguishing NSCLC subtypes based on MR images. Meanwhile, the Support Vector Machine (SVM) classifier applied to the same dataset achieved 0.75 accuracy [45]. It can be hypothesized that applying the SVM algorithm to the FOS/SOS/FDTA dataset may yield even higher classification accuracy—potentially comparable to the 0.80 accuracy and 0.82 AUC reported for CT images [46]—though this warrants further investigation.

While SOS texture features alone may not always yield optimal classification accuracy [31,32], they often complement other approaches and have been used as prognostic imaging biomarkers. Radiomics analysis of texture features has contributed not only to subtype differentiation but also to clinical decision-making and early-stage cancer detection [36,47]. For example, SOS texture features derived from CT images using GLCM have been associated with survival outcomes in patients with NSCLC [48]. Aerts et al. [12] extended the application of radiomics to predict treatment response in lung cancer, showing that texture features extracted from pre-treatment scans correlated with patient outcomes following radiotherapy or chemotherapy. Bracci et al. [47] explored the role of CT-derived texture features in predicting programmed death-ligand 1 (PD-L1) expression, identifying radiomic markers from GLCM, GLRLM, NGTDM, and GLSZM that differentiated patient subgroups with distinct treatment needs. Similarly, Ganeshan et al. [48] found correlations between texture features extracted using FOS and GLCM approaches and the histopathological characteristics of NSCLC, noting greater heterogeneity in malignant versus benign tumors. This distinction underscores the potential of texture analysis as a valuable diagnostic tool. Building on previous research into the clinical relevance of MR image radiomics, the findings of this study suggest that fractal-based texture features hold promise as non-invasive imaging biomarkers for NSCLC subtype differentiation. However, further research is needed to elucidate the relationship between FDTA features and detailed histopathological findings, treatment response, prognosis, and survival outcomes. Such insights could support the development of more personalized diagnostic and therapeutic strategies in NSCLC management.

## 5. Conclusions

The inclusion of fractal descriptors in the classification of lung MR images enhanced the differentiation between ADC and SCC subtypes. The overall improvement in classification metrics with the addition of fractal descriptors may result from the ability of FDTA features to capture structural heterogeneity and complexity not fully represented by FOS and SOS texture features alone. Utilizing the random forest algorithm for texture feature selection yielded higher classification performance than relying solely on features that were statistically different between ADC and SCC MR images. This suggests potential interrelationships among certain FOS, SOS, and FDTA features that warrant further investigation. The integration of FDTA into radiomic workflows improves the diagnostic performance of machine learning models—particularly kNN—in differentiating ADC and SCC in MR images. These findings support the potential of fractal-based imaging biomarkers as non-invasive, reproducible tools that may contribute to more personalized management strategies in NSCLC.

## Figures and Tables

**Table 1:** Texture feature extracted using first-order statistics (FOS), second-order statistics (SOS), and fractal dimension texture analysis (FDTA).

FOS	SOS					FDTA
	GLCM	GLDM	GLRLM	GLSZM	NGTDM	
Mean	Autocorrelation	SDE (Small Dependence Emphasis)	SRE (Short Run Emphasis)	SAE (Small Area Emphasis)	Busyness	FD (Fractal Dimension)
Median	CP (Cluster Prominence)	LDE (Large Dependence Emphasis)	LRE (Long Run Emphasis)	LAE (Large Area Emphasis)	Coarseness	Lacunarity
Skewness	CS (Cluster Shade)	GLN (Gray-Level Non-Uniformity)	GLN (Gray-Level Non-Uniformity)	GLN (Gray-Level Non-Uniformity)	Complexity	FSVI (Fractal Scale Variability Index)
Kurtosis	CT (Cluster Tendency)	DN (Dependence Non-Uniformity)	GLNN (Gray-Level Non-Uniformity Normalized)	GLNN (Gray-Level Non-Uniformity Normalized)	Contrast	
Energy	Contrast	DNN (Dependence Non-Uniformity Normalized)	RLN (Run Length Non-Uniformity)	SZN (Size Zone Non-Uniformity)	Strength	
Entropy	Correlation	GLV (Gray-Level Variance)	RLNN (Run Length Non-Uniformity Normalized)	SZNN (Size Zone Non-Uniformity Normalized)		
Min (Minimum)	DA (Difference Average)	DV (Dependence Variance)	RP (Run Percentage)	ZP (Zone Percentage)		
Max (Maximum)	DE (Difference Entropy)	DE (Dependence Entropy)	GLV (Gray-Level Variance)	GLV (Gray-Level Variance)		
10th Percentile	DV (Difference Variance)	LGLE (Low Gray-Level Emphasis)	RV (Run Variance)	ZV (Zone Variance)		
90th Percentile	ID (Inverse Difference)	HGLE (High Gray-Level Emphasis)	RE (Run Entropy)	ZE (Zone Entropy)		
IR (Interquartile Range)	IDN (Inverse Difference Normalized)	SDLGLE (Small Dependence Low Gray-Level Emphasis)	LGLRE (Low Gray-Level Run Emphasis)	LGLZE (Low Gray-Level Zone Emphasis)		
Range	IMC1 (Informational Measure of Correlation 1)	SDHGLE (Small Dependence High Gray-Level Emphasis)	HGLRE (High Gray-Level Run Emphasis)	HGLZE (High Gray-Level Zone Emphasis)		
MAD (Mean Absolute Deviation)	IMC2 (Informational Measure of Correlation 2)	LDLGLE (Large Dependence Low Gray-Level Emphasis)	LRLGLE (Long Run Low Gray-Level Emphasis)	SALGLE (Small Area Low Gray-Level Emphasis)		
rMAD (Robust Mean Absolute Deviation)	IDM (Inverse Difference Moment)	LDHGLE (Large Dependence High Gray-Level Emphasis)	LRHGLE (Long Run High Gray-Level Emphasis)	SAHGLE (Small Area High Gray-Level Emphasis)		
RMS (Root Mean Squared)	IDMN (Inverse Difference Moment Normalized)		SRLGLE (Short Run Low Gray-Level Emphasis)	LALGLE (Large Area Low Gray-Level Emphasis)		
Uniformity	JA (Joint Average)		SRHGLE (Short Run High Gray-Level Emphasis)	LAHGLE (Large Area High Gray-Level Emphasis)		
Variance	JEn (Joint Energy)					
	JEnt (Joint Entropy)					
	IV (Inverse Variance)					
	MP (Maximum Probability)					
	SE (Sum Entropy)					
	SS (Sum Squares)					

**Table 2:** Texture features (median and range (lower quartile (Q1); upper quartile (Q3)) extracted using first-order statistics (FOS) from MR images, which differed between adenocarcinoma (ADC) and squamous cell carcinoma (SCC).

FOS Texture Features	ADC	SCC	*p*-Value
Mean	0.46 (0.39; 0.54) ^a^	0.44 (0.32; 0.51) ^b^	0.01
Median	0.58 (0.48; 0.66) ^a^	0.54 (0.39; 0.62) ^b^	0.003
Kurtosis	3.19 (2.55; 4.69) ^a^	3.97 (3.01; 5.24) ^b^	0.001
Min	−1.68 (−1.98; −1.41) ^a^	−1.82 (−2.22; −1.61) ^b^	0.004
10th Percentile	−0.48 (−0.61; −0.23) ^a^	−0.34 (−0.55; −0.15) ^b^	0.01
90th Percentile	1.17 (1.01; 1.32) ^a^	1.05 (0.93; 1.19) ^b^	<0.0001
IR	0.83 (0.58; 1.05) ^a^	0.62 (0.48; 0.92) ^b^	0.0003
MAD	0.52 (0.40; 0.60) ^a^	0.42 (0.34; 0.54) ^b^	0.0003
rMAD	0.37 (0.26; 0.44) ^a^	0.28 (0.22; 0.40) ^b^	0.0006
RMS	0.82 (0.71; 0.87) ^a^	0.74 (0.66; 0.81) ^b^	<0.0001
Variance	0.42 (0.29; 0.55) ^a^	0.30 (0.22; 0.46) ^b^	0.0007

Superscript letters (^a^,^b^) indicate differences between non-small cell lung cancer (NSCLC) subtypes. Statistical significance was set at *p* < 0.05.

**Table 3:** Texture features (median and range (lower quartile (Q1); upper quartile (Q3)) extracted using Gray-Level Co-occurrence Matrix (GLCM) belong to second-order statistics (SOS) from MR images, which differed between adenocarcinoma (ADC) and squamous cell carcinoma (SCC).

GLCM Texture Features	ADC	SCC	*p*-Value
CP	1.19 (1.12; 1.22) ^a^	1.15 (1.01; 1.22) ^b^	0.008
CS	−0.67 (−0.70; −0.60) ^a^	−0.65 (−0.69; −0.55) ^b^	0.02
Correlation	0.90 (0.88; 0.91) ^a^	0.89 (0.87; 0.91) ^b^	0.02
IMC2	0.81 (0.77; 0.84) ^a^	0.80 (0.74; 0.82) ^b^	0.005

Superscript letters (^a^,^b^) indicate differences between non-small cell lung cancer (NSCLC) subtypes. Statistical significance was set at *p* < 0.05.

**Table 4:** Texture feature (median and range (lower quartile (Q1); upper quartile (Q3)) extracted using Gray-Level Dependence Matrix (GLDM) belong to second-order statistics (SOS) from MR images, which differed between adenocarcinoma (ADC) and squamous cell carcinoma (SCC).

GLDM Texture Feature	ADC	SCC	*p*-Value
SDHGLE	0.046 (0.045; 0.047) ^a^	0.047 (0.046; 0.048) ^b^	0.0009

Superscript letters (^a^,^b^) indicate differences between non-small cell lung cancer (NSCLC) subtypes. Statistical significance was set at *p* < 0.05.

**Table 5:** Texture features (median and range (lower quartile (Q1); upper quartile (Q3)) extracted using Gray-Level Run Length Matrix (GLRLM) belong to second-order statistics (SOS) from MR images, which differed between adenocarcinoma (ADC) and squamous cell carcinoma (SCC).

GLRLM Texture Features	ADC	SCC	*p*-Value
SRE	0.10 (0.08; 0.11) ^a^	0.11 (0.08; 0.12) ^b^	0.01
GLNN	0.53 (0.52; 0.54) ^a^	0.52 (0.51; 0.54) ^b^	0.0002
GLV	0.237 (0.228; 0.242) ^a^	0.241 (0.232; 0.247) ^b^	0.0002
LGLRE	0.71 (0.69; 0.74) ^a^	0.70 (0.66; 0.73) ^b^	0.0002
HGLRE	2.16 (2.05; 2.23) ^a^	2.21 (2.10; 2.35) ^b^	0.0002
SRLGLE	0.08 (0.07; 0.10) ^a^	0.09 (0.07; 0.11) ^b^	0.04
SRHGLE	0.13 (0.10; 0.16) ^a^	0.14 (0.10; 0.20) ^b^	0.04

Superscript letters (^a^,^b^) indicate differences between non-small cell lung cancer (NSCLC) subtypes. Statistical significance was set at *p* < 0.05.

**Table 6:** Texture features (median and range (lower quartile (Q1); upper quartile (Q3)) extracted using Gray-Level Size Zone Matrix (GLSZM) belong to second-order statistics (SOS) from MR images, which differed between adenocarcinoma (ADC) and squamous cell carcinoma (SCC).

GLSZM Texture Features	ADC	SCC	*p*-Value
GLN	5.29 (2.50; 11.15) ^a^	7.96 (4.14; 17.85) ^b^	0.0006
GLNN	0.65 (0.56; 0.76) ^a^	0.72 (0.61; 0.80) ^b^	0.002
SZN	1.00 (1.00; 1.42) ^a^	1.21 (1.00; 1.93) ^b^	0.002
SZNN	0.14 (0.09; 0.25) ^a^	0.11 (0.08; 0.18) ^b^	0.01
ZP	0.0006 (0.0005; 0.0011) ^a^	0.0007 (0.0005; 0.0015) ^b^	0.04
GLV	0.17 (0.12; 0.22) ^a^	0.14 (0.10; 0.19) ^b^	0.002
ZE	2.83 (2.00; 3.71) ^a^	3.18 (2.54; 4.04) ^b^	0.005
LGLZE	0.83 (0.75; 0.89) ^a^	0.88 (0.80; 0.92) ^b^	0.002
HGLZE	1.68 (1.43; 2.00) ^a^	1.50 (1.33; 1.79) ^b^	0.002
SALGLE	0.08 (0.002; 0.16) ^a^	0.12 (0.02; 0.20) ^b^	0.04

Superscript letters (^a^,^b^) indicate differences between non-small cell lung cancer (NSCLC) subtypes. Statistical significance was set at *p* < 0.05.

**Table 7:** Texture features (median and range (lower quartile (Q1); upper quartile (Q3)) extracted using Neighboring Gray-Tone Difference Matrix (NGTDM) belong to second-order statistics (SOS) from MR images, which differed between adenocarcinoma (ADC) and squamous cell carcinoma (SCC).

NGTDM Texture Features	ADC	SCC	*p*-Value
Coarseness	0.006 (0.004; 0.008) ^a^	0.005 (0.003; 0.007) ^b^	0.03
Strength	0.006 (0.003; 0.008) ^a^	0.005 (0.003; 0.007) ^b^	0.03

Superscript letters (^a^,^b^) indicate differences between non-small cell lung cancer (NSCLC) subtypes. Statistical significance was set at *p* < 0.05.

**Table 8:** Texture features (median and range (lower quartile (Q1); upper quartile (Q3)) extracted using fractal dimension texture analysis (FDTA) from MR images, which differed between adenocarcinoma (ADC) and squamous cell carcinoma (SCC).

FDTA Texture Features	ADC	SCC	*p*-Value
FD	2.428 (2.422; 2.434) ^a^	2.433 (2.426; 2.445) ^b^	<0.0001
Lacunarity	0.32 (0.29; 0.35) ^a^	0.30 (0.27; 0.34) ^b^	0.002

Superscript letters (^a^,^b^) indicate differences between non-small cell lung cancer (NSCLC) subtypes. Statistical significance was set at *p* < 0.05.

## Data Availability

In order to facilitate the replication of the experiments presented in this work, the dataset (data set location: https://doi.org/10.5281/zenodo.15907912 (accessed on 15 July 2025)) and the experimental source code are made available to the public under an open license.

## Supplementary Materials

The following supporting information can be downloaded at: https://www.mdpi.com/article/10.3390/jcm14165776/s1, Table S1: Texture features extracted using first-order statistics (FOS) from MR images representing adenocarcinoma (ADC) and squamous cell carcinoma (SCC). Table S2: Texture features extracted using Gray-Level Co-occurrence Matrix (GLCM) belong to second-order statistics (SOS) from MR images representing adenocarcinoma (ADC) and squamous cell carcinoma (SCC). **Table S3**: Texture features extracted using Gray-Level Dependence Matrix (GLDM) belong to second-order statistics (SOS) from MR images representing adenocarcinoma (ADC) and squamous cell carcinoma (SCC). Table S4: Texture features extracted using Gray-Level Run Length Matrix (GLRLM) belong to second-order statistics (SOS) from MR images representing adenocarcinoma (ADC) and squamous cell carcinoma (SCC). **Table S5**: Texture features extracted using Gray-Level Size Zone Matrix (GLSZM) belong to second-order statistics (SOS) from MR images representing adenocarcinoma (ADC) and squamous cell carcinoma (SCC). Table S6: Texture features extracted using Neighboring Gray-Tone Difference Matrix (NGTDM) belong to second-order statistics (SOS) from MR images representing adenocarcinoma (ADC) and squamous cell carcinoma (SCC). Table S7: Texture features extracted using fractal dimension texture analysis (FDTA) from MR images representing adenocarcinoma (ADC) and squamous cell carcinoma (SCC). Table S8. Summary of texture features used for the non-small cell lung cancer (NSCLC) subtype classification.
