# Supplementary material for: Application of Fractal Radiomics and Machine Learning for Differentiation of Non-Small Cell Lung Cancer Subtypes on PET/MR Images
Source: J Clin Med. 2025 Aug 15;14(16):5776. doi: 10.3390/jcm14165776 (PMC12386986; doi:10.3390/jcm14165776)
Supplement: Supplementary file 1 [file jcm-14-05776-s001.zip › jcm-3789973-supplementary.pdf]

Journal of Clinical Medicine

Supplementary Data for

Application of Fractal Radiomics and Machine Learning for Classification of Non–Small Cell Lung Cancer Subtypes on MR Images

Ewelina Bębas [1], Konrad Pauk [2], Jolanta Pauk [1], Kristina Daunoravičienė [3], Małgorzata Mojsak [4], Marcin Hładuński [4], Małgorzata Domino [5,*] and Marta Borowska [1,*]

[1] Institute of Biomedical Engineering, Bialystok University of Technology, 15-351 Białystok, Poland; ewebeb7@gmail.com (E.B.); j.pauk@pb.edu.pl (J.P.)
[2] Faculty of Medicine, Warsaw Medical University, 02-091 Warszawa, Poland; konrad.pauk@gmail.com
[3] Department of Biomechanical Engineering, Vilnius Gediminas Technical University, 10223 Vilniaus, Lithuania; kristina.daunoraviciene@vlniustech.lt
[4] Laboratory of Molecular Imaging, Medical University of Białystok, 15-089 Białystok, Poland; malgorzata.mojsak@umb.edu.pl (M.M.); hladunski.marcin@gmail.com (M.H.)
[5] Department of Large Animal Diseases and Clinic, Institute of Veterinary Medicine, Warsaw University of Life Science, 02-787 Warszawa, Poland
* Correspondence: malgorzata_domino@sggw.edu.pl (M.D.); m.borowska@pb.edu.pl (M.B.)

Table S1. Texture features (median and range (lower quartile (Q1); upper quartile (Q3)) extracted using first–order statistics (FOS) from MR images representing adenocarcinoma (ADC) and squamous cell carcinoma (SCC).

FOS texture features	ADC	SCC	*p*-value
Mean	0.46 (0.39; 0.54)[a]	0.44 (0 32; 0.51)[b]	**0.01**
Median	0.58 (0.48; 0.66)[a]	0.54 (0,39; 0,62)[b]	**0.003**
Skewness	-0.68 (-1.18;-0.31)	-0.77 (-1.09;-0.39)	0.64
Kurtosis	3.19 (2.55; 4.69)[a]	3.97 (3.01; 5.24)[b]	**0.001**
Energy	7577 (5504; 11865)	7203 (5302; 11073)	0.40
Entropy	0.74 (0.61; 0.85)	0.71 (0.57; 0.83)	0.08
Min	-1.68 (-1.98; -1.41)[a]	-1.82 (-2.22; -1.61)[b]	**0.004**
Max	1.90 (1.69; 2.26)	1.87 (1.66; 2.24)	0.85
10 Percentile	-0.48 (-0.61; -0.23)[a]	-0.34 (-0.55; -0.15)[b]	**0.01**
90 Percentile	1.17 (1.01; 1.32)[a]	1.05 (0.93; 1.19)[b]	**<0.0001**
IR	0.83 (0.58; 1.05)[a]	0.62 (0.48; 0 92)[b]	**0.0003**
Range	3.65 (3.29; 4.10)	3.62 (3.34; 4.35)	0.36
MAD	0.52 (0.40; 0.60)[a]	0.42 (0.34; 0.54)[b]	**0.0003**
rMAD	0.37 (0.26; 0.44)[a]	0.28 (0.22; 0.40)[b]	**0.0006**
RMS	0.82 (0.71; 0.87)[a]	0.74 (0.66; 0.81)[b]	**<0.0001**
Uniformity	0.67 (0.60; 0.74)	0.69 (0.61; 0.77)	0.08
Variance	0.42 (0.29; 0.55)[a]	0.30 (0.22; 0.46)[b]	**0.0007**

Superscripts letters (a–b) indicate differences between non–small cell lung cancer (NSCLC) subtypes. Statistical significance was set at $p < 0.05$ and highlighted using bold font.

Table S2. Texture features (median and range (lower quartile (Q1); upper quartile (Q3)) extracted using Gray–Level Co–occurrence Matrix (GLCM) belong to second-order statistics (SOS) from MR images representing adenocarcinoma (ADC) and squamous cell carcinoma (SCC).

GLCM texture features	ADC	SCC	*p*-value
Autocorrelation	3.38 (3.19; 3.56)	3.43 (3.23; 3.61)	0.11
CP	1.19 (1.12; 1.22)[a]	1.15 (1.01; 1.22)[b]	**0.008**
CS	-0.67 (-0.70; -0.60)[a]	-0.65 (-0.69; -0.55)[b]	**0.02**
CT	0.62 (0.47; 0.74)	0.56 (0.42; 0.71)	0.08
Contrast	0.03 (0.02; 0.04)	0.03 (0.02; 0.04)	0.86
Correlation	0.90 (0.88; 0.91)[a]	0.89 (0.87; 0.91)[b]	**0.02**
DA	0.03 (0.02; 0.04)	0.03 (0.02; 0.04)	0.86
DE	0.20 (0.15; 0.24)	0.19 (0.15; 0.24)	0.86
DV	0.03 (0.02; 0.04)	0.03 (0.02; 0.04)	0.85
ID	0.98 (0.98; 0.99)	0.98 (0.98; 0.99)	0.86
IDN	0.99 (0.99; 0.99)	0.99 (0.99; 0.99)	0.86
IMC1	-0.73 (-0.77; -0.68)	-0.73 (-0.76; -0.68)	0.11
IMC2	0.81 (0.77; 0.84)[a]	0.80 (0.74; 0.82)[b]	**0.005**
IDM	0.98 (0.98; 0.99)	0.98 (0.98; 0.99)	0.86
IDMN	0.99 (0.99; 1.00)	0.99 (0.99; 1.00)	0.86
JA	1.80 (1.74; 1.86)	1.82 (1.75; 1.87)	0.10
JEn	0.65 (0.57; 0.73)	0.67 (0.59; 0.76)	0.14
JEnt	0.93 (0.76; 1.04)	0.89 (0.68; 1.04)	0.19
IV	0.03 (0.02; 0.04)	0.03 (0.02; 0.04)	0.86
MP	0.78 (0.72; 0.84)	0.80 (0.73; 0.86)	0.13
SE	0.89 (0.73; 1.02)	0.85 (0.66; 1.00)	0.17
SS	0.16 (0.12; 0.19)	0.15 (0.11; 0.19)	0.10

Superscripts letters (a–b) indicate differences between non–small cell lung cancer (NSCLC) subtypes. Statistical significance was set at $p < 0.05$ and highlighted using bold font.

Table S3. Texture features (median and range (lower quartile (Q1); upper quartile (Q3)) extracted using Gray–Level Dependence Matrix (GLDM) belong to second-order statistics (SOS) from MR images representing adenocarcinoma (ADC) and squamous cell carcinoma (SCC).

GLDM texture features	ADC	SCC	*p*-value
SDE	0.01 (0.01; 0.02)	0.01 (0.01; 0.02)	0.91
LDE	75.6 (74.1; 77.0)	75.6 (74.5; 77.2)	0.56
GLN	7251 (4950; 14021)	10262 (5982; 16098)	0.07
DN	8216 (5566; 15645)	10756 (6328; 17186)	0.15
DNN	0.74 (0.67;.0.80)	0.74 (0.69; 0.81)	0.56
GLV	0.17 (0.13; 0.20)	0.16 (0.12; 0.19)	0.08
DV	0.98 (0.77; 1.23)	0.96 (0.74; 1.19)	0.78
DE	1.53 (1.28; 1.81)	1.49 (1.17; 1.77)	0.29
LGLE	0.41 (0.36; 0.46)	0.39 (0.35; 0.45)	0.08
HGLE	3.37 (3.16; 3.55)	3.42 (3.22; 3.60)	0.08
SDLGLE	0.01 (0.01; 0.01)	0.01 (0.01; 0.01)	0.28
SDHGLE	0.046 (0.045; 0.047)[a]	0.047 (0.046; 0.048)[b]	**0.0009**
LDLGLE	29.2 (26.6; 32.1)	28.2 (25.3; 31.6)	0.08
LDHGLE	260.7 (242.7; 276.9)	264.6 (246.0; 283.0)	0.15

Superscripts letters (a–b) indicate differences between non–small cell lung cancer (NSCLC) subtypes. Statistical significance was set at $p < 0.05$ and highlighted using bold font.

Table S4. Texture features (median and range (lower quartile (Q1); upper quartile (Q3)) extracted using Gray–Level Run Length Matrix (GLRLM) belong to second-order statistics (SOS) from MR images representing adenocarcinoma (ADC) and squamous cell carcinoma (SCC).

GLRLM texture features	ADC	SCC	*p*-value
SRE	0.10 (0.08; 0.11)[a]	0.11 (0.08; 0.12)[b]	**0.01**
LRE	1314 (781; 2609)	1380 (874; 2591)	0.64
GLN	280.5 (208.3; 405.8)	325.6 (223.7; 427.2)	0.08
GLNN	0.53 (0.52; 0.54)[a]	0.52 (0.51; 0.54)[b]	**0.0002**
RLN	25.9 (19.0; 35.1)	27.4 (21.3; 37.8)	0.10
RLNN	0.05 (0.04; 0.06)	0.05 (0.04; 0.06)	0.68
RP	0.05 (0.03; 0.06)	0.05 (0.03; 0.05)	0.60
GLV	0.237 (0.228; 0.242)[a]	0.241 (0.232; 0.247)[b]	**0.0002**
RV	763 (457; 1579)	816 (494; 1661)	0.70
RE	5.56 (5.30; 5.86)	5.65 (5.41; 5.87)	0.30
LGLRE	0.71 (0.69; 0.74)[a]	0.70 (0.66; 0.73)[b]	**0.0002**
HGLRE	2.16 (2.05; 2.23)[a]	2.21 (2.10; 2.35)[b]	**0.0002**
LRLGLE	389 (265; 711)	387 (255; 746)	0.75
LRHGLE	5091 (2888; 10116)	5318 (3301; 10225)	0.64
SRLGLE	0.08 (0.07; 0.10)[a]	0.09 (0.07; 0.11)[b]	**0.04**
SRHGLE	0.13 (0.10; 0.16)[a]	0.14 (0.10; 0.20)[b]	**0.04**

Superscripts letters (a–b) indicate differences between non–small cell lung cancer (NSCLC) subtypes. Statistical significance was set at $p < 0.05$ and highlighted using bold font.

Table S5. Texture features (median and range (lower quartile (Q1); upper quartile (Q3)) extracted using Gray–Level Size Zone Matrix (GLSZM) belong to second-order statistics (SOS) from MR images representing adenocarcinoma (ADC) and squamous cell carcinoma (SCC).

GLSZM texture features	ADC	SCC	*p*-value
SAE	0.11 (0.002; 0.20)	0.14 (0.02; 0.22)	0.09
LAE	12560000 (4759000; 27150000)	11860000 (4595000; 24920000)	0.55
GLN	5.29 (2.50; 11.15)[a]	7.96 (4.14; 17.85)[b]	**0.0006**
GLNN	0.65 (0.56; 0.76)[a]	0.72 (0.61; 0.80)[b]	**0.002**
SZN	1.00 (1.00; 1.42)[a]	1.21 (1.00; 1.93)[b]	**0.002**
SZNN	0.14 (0.09; 0.25)[a]	0.11 (0.08; 0.18)[b]	**0.01**
ZP	0.0006 (0.0005; 0.0011)[a]	0.0007 (0.0005; 0.0015)[b]	**0.04**
GLV	0.17 (0.12; 0.22)[a]	0.14 (0.10; 0.19)[b]	**0.002**
ZV	7065000 (3511000; 20930000)	6689000 (3514000; 20190000)	0.90
ZE	2.83 (2.00; 3.71)[a]	3.18 (2.54; 4.04)[b]	**0.005**
LGLZE	0.83 (0.75; 0.89)[a]	0.88 (0.80; 0.92)[b]	**0.002**
HGLZE	1.68 (1.43; 2.00)[a]	1.50 (1.33; 1.79)[b]	**0.002**
SALGLE	0.08 (0.002; 0.16)[a]	0.12 (0.02; 0.20)[b]	**0.04**
SAHGLE	0.13 (0.002; 0.24)	0.16 (0.02; 0.25)	0.13
LALGLE	4072000 (1456000; 7722000)	3458000 (1424000; 7034000)	0.38
LAHGLE	47600000 (18440000; 103900000)[a]	44220000 (17390000; 93170000)[b]	0.59

Superscripts letters (a–b) indicate differences between non–small cell lung cancer (NSCLC) subtypes. Statistical significance was set at $p < 0.05$ and highlighted using bold font.

Table S6. Texture features (median and range (lower quartile (Q1); upper quartile (Q3)) extracted using Neighbouring Gray–Tone Difference Matrix (NGTDM) belong to second-order statistics (SOS) from MR images representing adenocarcinoma (ADC) and squamous cell carcinoma (SCC).

NGTDM texture features	ADC	SCC	*p*-value
Busyness	64.6 (46.1; 102.9)	69.9 (48.7; 111.7)	0.18
Coarseness	0.006 (0.004; 0.008)[a]	0.005 (0.003; 0.007)[b]	**0.03**
Complexity	0.03 (0.02; 0.04)	0.03 (0.02; 0.04)	0.86
Contrast	0.005 (0.003; 0.008)	0.005 (0.003; 0.007)	0.34
Strength	0.006 (0.003; 0.008)[a]	0.005 (0.003; 0.007)[b]	**0.03**

Superscripts letters (a–b) indicate differences between non–small cell lung cancer (NSCLC) subtypes. Statistical significance was set at $p < 0.05$ and highlighted using bold font.

Table S7. Texture features (median and range (lower quartile (Q1); upper quartile (Q3)) extracted using fractal dimension texture analysis (FDTA) from MR images representing adenocarcinoma (ADC) and squamous cell carcinoma (SCC).

FDTA texture features	ADC	SCC	*p*-value
FD	2.428 (2.422; 2.434)[a]	2.433 (2.426; 2.445)[b]	**<0.0001**
Lacunarity	0.32 (0.29; 0.35)[a]	0.30 (0.27; 0.34)[b]	**0.002**
FSVI	-0.10 (-0.11; -0.08)	-0.09 (-0.11; -0.07)	0.07

Superscripts letters (a–b) indicate differences between non–small cell lung cancer (NSCLC) subtypes. Statistical significance was set at $p < 0.05$ and highlighted using bold font.

Table S8. Summary of texture features used for the non–small cell lung cancer (NSCLC) subtypes classification.

Approach	Texture features	Differed features in FOS/SOS/FDTA data set	Selected features from FOS/SOS data set	Selected features from FOS/SOS/FDTA data set
**FOS**	Mean	X	X	X
	Median	X	X	X
	Skewness*,**	-	X	X
	Kurtosis	X	X	X
	Energy*,**	-	X	X
	Min	X	X	X
	Max**	-	-	X
	10 Percentile	X	X	X
	90 Percentile	X	X	X
	IR	X	X	X
	Range*,**	-	X	X
	MAD	X	X	X
	rMAD	X	X	X
	RMS	X	X	X
	Variance	X	X	X
**SOS (GLCM)**	CP	X	-	-
	CS	X	-	-
	Correlation	X	X	-
	IMC1*,**	-	X	X
	IMC2	X	-	X
**SOS (GLDM)**	GLN*,**	-	X	X
	DN*,**	-	X	X
	SDHGLE	X	X	X
**SOS (GLRLM)**	SRE	X	X	X
	GLN*,**	-	X	X
	GLNN	X	X	X
	RLN*,**	-	X	X
	GLV	X	X	X
	RE*,**	-	X	X
	LGLRE	X	X	X
	HGLRE	X	X	X
	SRLGLE	X	X	X
	SRHGLE	X	X	X
**SOS (GLSZM)**	GLN	X	X	-
	GLNN	X	-	-
	SZN	X	-	-
	SZNN	X	-	-
	ZP	X	X	X
	GLV	X	-	-
	ZE	X	-	-
	LGLZE	X	X	-
	HGLZE	X	-	-
	SALGLE	X	-	-
**SOS (NGTDM)**	Busyness*	-	X	-
	Coarseness	X	X	X
	Complexity**	-	-	X
	Strength	X	X	X
**FDTA**	FD	X	-	X
	Lacunarity	X	-	X
	FSVI**	-	-	X
**n**		37	35	37

(X) texture features used in classification; (-) texture features not used in classification; (*,**) texture features that did not differ between ADC and SCC but were selected using the random forests algorithm for NSCLC subtypes classification from (*) FOS/SOS and (**) FOS/SOS/FDTA data sets.
